# Pedunculopontine Nucleus Gamma Band Activity-Preconscious Awareness, Waking, and REM Sleep

**DOI:** 10.3389/fneur.2014.00210

**Published:** 2014-10-20

**Authors:** Francisco J. Urbano, Stasia M. D’Onofrio, Brennon R. Luster, Paige B. Beck, James Robert Hyde, Veronica Bisagno, Edgar Garcia-Rill

**Affiliations:** ^1^IFIBYNE & ININFA-CONICET, University of Buenos Aires, Buenos Aires, Argentina; ^2^Center for Translational Neuroscience, Department of Neurobiology and Developmental Sciences, University of Arkansas for Medical Sciences, Little Rock, AR, USA

**Keywords:** bipolar disorder, calcium/calmodulin-dependent protein kinase II, cyclic adenosine monophosphate, leptin, neuronal calcium sensor, N- and P/Q-type calcium channels, schizophrenia

## Abstract

The pedunculopontine nucleus (PPN) is a major component of the reticular activating system (RAS) that regulates waking and REM sleep, states of high-frequency EEG activity. Recently, we described the presence of high threshold, voltage-dependent N- and P/Q-type calcium channels in RAS nuclei that subserve gamma band oscillations in the mesopontine PPN, intralaminar parafascicular nucleus (Pf), and pontine subcoeruleus nucleus dorsalis (SubCD). Cortical gamma band activity participates in sensory perception, problem solving, and memory. Rather than participating in the temporal binding of sensory events as in the cortex, gamma band activity in the RAS may participate in the processes of preconscious awareness, and provide the essential stream of information for the formulation of many of our actions. That is, the RAS may play an early permissive role in volition. Our latest results suggest that (1) the manifestation of gamma band activity during waking may employ a separate intracellular pathway compared to that during REM sleep, (2) neuronal calcium sensor (NCS-1) protein, which is over expressed in schizophrenia and bipolar disorder, modulates gamma band oscillations in the PPN in a concentration-dependent manner, (3) leptin, which undergoes resistance in obesity resulting in sleep dysregulation, decreases sodium currents in PPN neurons, accounting for its normal attenuation of waking, and (4) following our discovery of electrical coupling in the RAS, we hypothesize that there are cell clusters within the PPN that may act in concert. These results provide novel information on the mechanisms controlling high-frequency activity related to waking and REM sleep by elements of the RAS.

## Background

Why is the brainstem involved in the regulation of so many functions? One line of thought is that we must dissect out the foci or nodes responsible for widely disparate functions. However, this assumes that specific regions are involved in different functions. Instead, the evidence is overwhelming that the same structures are involved in multiple, quite disparate functions. Therefore, a critical question is what are the mechanisms that subserve so many different behavioral states, the initiation and maintenance of wakefulness, the facilitating motor control, the modulation of mood, as well as influencing a number of homeostatic systems? An alternative hypothesis is that these nuclei generate the appropriate carrier frequencies that underlie multiple processes. That is, these nodes may contain multiple cell types but they act in concert to give rise to the required underlying large-scale signals appropriate for each function. This suggests that it is the heterogenic cell groups or clusters, i.e., of multiple transmitter types, within nodes that may provide more valuable information.

Two processes involved in large-scale communication are coherence, signals mirrored by neurons across different areas, and frequency, the specific firing patterns required to transmit population dynamics across regions. The former process is modulated by electrical coupling while the latter process is mediated by membrane oscillations and synaptic connectivity (1, 2). The following review targets one component of the reticular activating system (RAS), the pedunculopontine nucleus (PPN), and its involvement in a large number of functions. This involvement is anchored by two recently discovered mechanisms, the presence of electrical coupling and generation of gamma band activity. It is these mechanisms that provide the coherence and appropriate frequencies to carry out its widespread effects.

In summary, the PPN is a major component of the RAS that regulates waking and REM sleep, two states of high-frequency EEG activity. In addition, the RAS modulates virtually every system in the brain, which has implications for (a) voluntary movement and movement disorders, (b) behavioral state and psychiatric disorders, and (c) synergy with other homeostatic systems like circadian, hormonal, and appetite control. We discuss the role of gamma band membrane oscillations that were recently described in every cell in this nucleus, the presence of cell clusters in the PPN, and how these impact a number of specific functions.

## Waking and Preconscious Awareness

When we awaken, blood flow to the thalamus and brainstem increases over the first 15 min. It is only later that there are increases in blood flow to the cortex (3). Yet, we wake up as ourselves, it does not take 15–20 min to figure out who we are. This suggests that subcortical regions have much to do with our sense of self (4). In addition, our highest functions are mediated by fast cortical oscillations, the 40 Hz rhythm, or gamma band oscillations. Yet gamma band activity has been described not only in the cortex, but also in the thalamus, hippocampus, cerebellum, basal ganglia, and now, in the RAS. Not only that, but these are not independent oscillations, they are coherent depending on the task at hand. Under some conditions, gamma activity in subcortical areas even precedes cortical gamma activity (5, 6).

What is the role of gamma band activity in the cortex? It is thought to participate in binding of perception, in the unification of different aspects of a sensory event. For example, a visual image has color, depth, structure, movement, and each of these properties is processed by a different cortical region. The coherent activation by gamma oscillations across these regions was proposed to provide the unification of all of these features, the binding of the sensory event to facilitate perception.

What is the role of gamma band activity in the RAS? We know that the RAS receives a constant stream of information from the senses, and also receives ongoing activity from within the brain. What is the unifying function of gamma band activity in the RAS? We proposed that the maintenance of gamma band activity in the RAS provides information for the process necessary to support a state capable of reliably assessing the world around us on a continuous basis. That is, it provides the mechanism for the process of preconscious awareness (1, 2).

The simple act of waking up now gains a much more complex role. It needs to integrate our world with ourselves, while we use other parts of our brains to formulate our plans and desires. As we will see, we may not be paying attention to some of these plans and desires, that is, we are not consciously paying attention to a mass of information that we nevertheless process preconsciously. As such, the RAS is involved in anonymously formulating movements and actions of which we are not consciously (but only preconsciously) aware. This expands the purview of the background of activity in the RAS as not only allowing afferent information to flow into the brain, but in establishing the background of activity on which we superimpose volition and free will.

Lesions of the PPN in the cat were found to reduce REM sleep events as well as ponto-geniculo-occipital (PGO) waves and REMs (7). Other studies using PPN lesions were performed in rats and were confirmed to affect sleep architecture, mainly increasing SWS and fractionating REM sleep episodes, especially disturbing transitions between SWS, REM sleep, and waking, but without major effects on waking duration (8, 9). Behavioral studies showed that excitotoxic lesions of the PPN impaired the acquisition of several learning tasks including, spatial navigation in the Morris water maze with a submerged platform (10), one-trial passive avoidance and two-way shuttle box active avoidance (11), externally cued reinforced bar pressing (12), delayed spatial win-shift task in an eight-arm radial maze (13), and spatial delayed matching- and non-matching-to-position in a T-maze (14, 15). In general, then, waking and REM sleep are disturbed but not eliminated, and disturbances in complex behaviors ensue when unilateral vs bilateral lesions are executed. This is not surprising when lesioning a homeostatic system with such global function as coordinating exteroceptive and internal information, and modulating responses to environmental conditions. Most studies have employed behavioral tasks that require only narrow responsiveness or ask limited questions about sleep architecture or behavioral responses, but few assess the types of activity that would entail preattentional functions and sensory gating. Below, we address studies on the P50 potential in human beings and P13 potential in the rat that are generated by the PPN that measure sensory gating or habituation to repetitive stimulation (16). This process is impaired in a number of neurological and psychiatric disorders. It is worth remembering that injections of GABA or GABA agonists into the PPN reduced and eliminated the vertex-recorded P13 potential (17). This sleep state-dependent waveform is a manifestation of PPN outputs through the intralaminar thalamus to the cortex.

## Volition and Free Will

Pioneering studies by Libet first showed that when people consciously set a goal to engage in a behavior, their conscious will to act begins “unconsciously” (18). These authors studied the readiness potential (RP), a negative DC shift present long before the execution of a voluntary movement. The RP is present at maximal amplitude at the vertex (19), that is, the RP is manifested over the same region as the midlatency auditory evoked P50 potential. The P50 potential is a sleep state-dependent waveform present during waking and REM sleep that is generated by PPN outputs to the intralaminar thalamus and the cortex in response to an auditory stimulus (16). The RP is not only decreased in Parkinson’s disease (PD), but stimulation of the PPN in PD patients implanted for deep brain stimulation (DBS), manifest the most significant changes in blood flow in the same region, that is, at the vertex (20). The use of PPN DBS was recently reviewed (21), and will not be reiterated here, however, the latest findings show that PPN DBS using continuous 40 Hz stimulation in an animal model of PD improved movement time in a delayed sensorimotor task and reduced 6-OHDS-induced rotational movements (22). It would be interesting to determine if the RP represents preparatory activity in the RAS and intralaminar thalamus.

Libet’s subjects were asked to move voluntarily, and to subjectively time the moment at which they felt the “will” to move as well as the onset of the actual movement. The RP began well before their conscious “will” to move so that Libet suggested that voluntary acts begin “unconsciously,” before there is subjective “conscious” awareness that a decision to act was initiated by the brain. This conclusion has been extrapolated to suggest that there is no free will, however, Libet suggested that, although the movement was indeed initiated “subconsciously,” it was subject to veto once it reached consciousness (23). This has been regarded as unsatisfactory and not answering the question of whether there is free will or not. The question is complex because so many factors influence the sense of volition such as the perception of time, the conditions under which the movement is executed and the perception of volition (24). We propose an alternative view by concluding that it is the interpretation of the results that assumes that the process preceding the movement is “unconscious.” There is no evidence that this is the case. The preparation for movement generates waking brain processes that are clearly related to the intent, and therefore should be labeled “preconscious,” not “subconscious.” That is, the conclusion of Libet’s study should have been “voluntary acts begin preconsciously, before there is subjective conscious awareness that a decision to act was initiated by the brain.” The RAS was proposed to mediate the process of preconscious awareness. The mechanism behind this process is gamma band activity.

## Gamma Band Activity

Gamma frequency oscillations are thought to participate in sensory perception, problem solving, and memory (25–30). Coherence at gamma band frequencies can occur at cortical (31) or thalamocortical levels (32). In addition, gamma band activity has been described in the hippocampus (33, 34), the cerebellum (35, 36), and the basal ganglia (37). As mentioned above, gamma band activity in the motor cortex lags behind coherent activity in subcortical structures (5, 6). This led to the suggestion that motor cortex gamma synchronization reflects a momentary arousal-related event for enabling the initiation of movement (38–40). That is, structures such as the RAS and thalamus may play an early permissive role in the control of voluntary movement. This again suggests that it would be worth determining if indeed the RP represents preparatory, arousal-related activity in the RAS and intralaminar thalamus.

Early studies described the presence of PPN neurons *in vivo* that fired at gamma frequencies. Extracellular recordings of PPN neurons *in vivo* identified six categories of thalamic projecting PPN cells distinguished by their firing properties relative to PGO wave generation (41). Some of these neurons had low rates of spontaneous firing (<10 Hz), but most had high rates of tonic firing (20–80 Hz) *in vivo*. It should be noted that these studies were performed in the absence of anesthesia but in a quiescent animal. PPN neurons are known to increase firing during REM sleep (REM-on), or both waking and REM sleep (Wake/REM-on), as well as waking only (Wake-on), but decrease during slow-wave sleep (SWS) (42–45), suggestive of increased excitation during activated states *in vivo*. The cat showed more waking-related activity while the rat manifested more REM sleep-related activity, however, it is not known if more active preparations, as opposed to these recordings performed during “quiet waking,” would exhibit higher frequency firing. Electrical stimulation of the PPN was found to potentiate the appearance of fast (20–40 Hz) oscillations in the cortical EEG *in vivo*, outlasting stimulation by 10–20 s (46). This suggests that the PPN can indeed modulate cortical high-frequency activity. More recently, identified cholinergic PPN neurons were found to fire in phase with cortical gamma oscillations (47). Most studies have shown fairly slow activity in PPN neurons (42, 48) not approaching the gamma range, however, this is likely due to the use of urethane anesthesia. These studies generate valuable information, but it must be remembered that procedures that decrease arousal are highly likely to decrease PPN cell activity. Moreover, even most *in vivo* studies in the absence of anesthetics tend to record PPN cell activity during quiescent states, i.e., “quiet waking,” which means the animal is inactive and unlikely to demonstrate the full capabilities of preattentional functions. Although difficult, the ideal protocols to study such functions may include sensory stimulation along with masking or priming stimuli calling for response inhibition as well as overt motor action. That is, a “normal” environment, in which there is continuous afferent information that requires the system to detect events without attending to them, is perhaps the ideal test system for preconscious awareness.

We discovered the presence of gamma band oscillations in three major centers of the RAS, (a) the PPN (49–51), (b) its major ascending target, the intralaminar parafascicular nucleus (Pf) (50, 52), and (c) its major descending target, the dorsal pontine subcoeruleus nucleus dorsalis (SubCD) (53). These were recently reviewed (1, 2). Briefly, all PPN and Pf neurons appear to oscillate at beta/gamma frequencies through P/Q- and N-type calcium channels, but in SubCD cells, beta/gamma band activity is mediated by sodium-dependent subthreshold oscillations. We found that these calcium channels were located on PPN and Pf dendrites and oscillate in synchrony with membrane oscillations (54, 55).

We also determined that the *maintenance* of gamma band activity is modulated by G proteins (56). This interesting mechanism suggests that tonic cholinergic input to inhibitory M2 receptors on PPN neurons is required in order to occupy G proteins. In doing so, the N- and P/Q-type calcium channels are not engaged by G proteins and are free to oscillate upon receiving input, presumably from sensory systems, onto their dendrites. This “permissive inhibition” of G proteins by tonic cholinergic input is a mechanism for the *maintenance* of gamma band activity (56). One question that arises, as these regions modulate both waking and REM sleep, are there differences in the gamma band activity generated during each of these two states?

## Waking vs REM sleep

The differences between gamma-band activity during waking and REM sleep are unknown. Why is this important? Because it is high frequency, especially beta/gamma band activity that drives not only our cognitive function during waking but also our dreams during REM sleep, two obviously different states of awareness. Our studies addressed the differential intracellular mechanisms assumed to subserve high-frequency activity during waking vs REM sleep as a prelude to the selective pharmacological modulation of these states. We know that the two states are differentially regulated in the PPN. Injections of glutamate into the PPN were shown to increase both waking and REM sleep, but injections of NMDA increased only waking, while injections of kainic acid (KA) increased only REM sleep (57–60). Moreover, the intracellular pathways mediating the two states appear to differ. For example, the CaMKII activation inhibitor, KN-93, microinjected into the PPN of freely moving rats resulted in decreased waking but not REM sleep (61). Increased ERK1/2 signaling in the PPN is associated with maintenance of sleep via suppression of waking (62) and activation of intracellular protein kinase A (PKA) in the PPN instead contributed to REM sleep recovery following REM sleep deprivation (63). These results suggest that waking is modulated by the CaMKII pathway, while REM sleep is modulated by the cAMP-PKA pathway.

**Figure 1:**
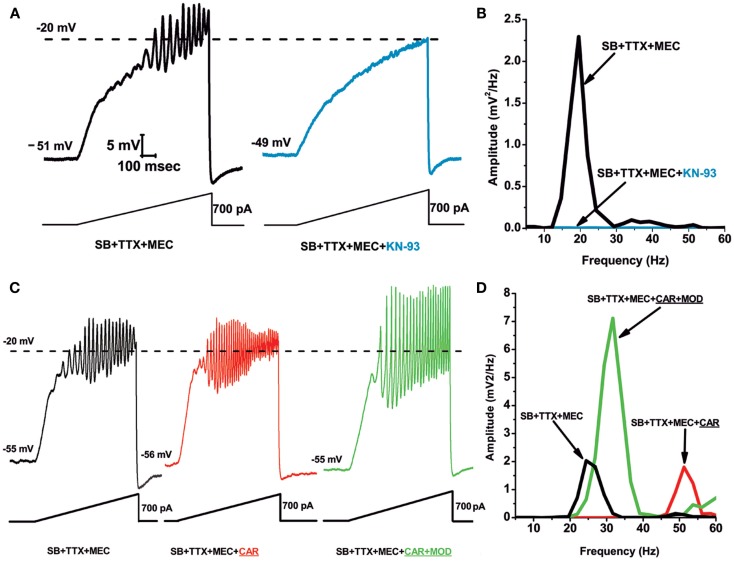
**Role of CaMKII in PPN membrane oscillations**. **(A)** Ramps induced oscillations in the beta range (black record). Following superfusion with KN-93 (10 μM) for 10 min, ramps no longer elicited oscillations (blue record). This suggests that beta/gamma oscillations in PPN neurons require the CaMKII pathway. **(B)** Power spectrum showing amplitude and frequency of ramp-induced oscillations before (black record, beta range) and after KN-93 (blue record, no oscillations). **(C)** Recordings of a PPN neuron before administration of CAR (30 μM) (black record), 20 min after continuous superfusion with CAR (red record), and 20 min after continuous superfusion with MOD and CAR (green record). **(D)** Power spectrum before CAR (black line, beta range), after CAR (red line, gamma range), and after CAR + MOD (green line, beat/gamma range). These results suggest that the stimulant MOD, which requires the CaMKII pathway, potentiates responses to cholinergic input to the PPN (64).

Figure 1 shows results demonstrating that the CaMKII pathway blocker KN-93 eliminated the ability of ramps to induce oscillations in PPN neurons (Figure 1A), an effect confirmed by the absence of oscillations in the power spectrum (Figure 1B). Recordings were carried out in the presence of synaptic blockers (SB), tetrodotoxin (TTX), and mecamylamine (MEC), a nicotinic receptor antagonist. These findings suggest that blocking the activation of CaMKII using KN-93 eliminated the ability of ramps to induce gamma band oscillations in PPN neurons. These data suggest that CaMKII is necessary for the manifestation of ramp-induced oscillations in at least some PPN neurons (64).

The effects of the stimulant modafinil (MOD) are dependent on CaMKII, since its effects are blocked by the CaMKII activation blocker KN-93 (65). Our studies using MOD extensively characterized its effects on (a) electrical coupling in the RAS and ILT, (b) established the concentration dependence of its effects *in vitro* and *in vivo*, (c) in animals and human beings, and (d) determined that its effects were blocked specifically by gap junction blockers (50, 66–69). MOD, which typically takes 10–20 min to induce changes in electrical coupling, by itself does not appear to change gamma band oscillation frequency or amplitude. However, MOD was found to enhance cholinergic agonist-induced oscillation amplitude. We used SB plus the nicotinic receptor antagonist MEC to tonically activate only muscarinic receptors using carbachol (CAR). Figures 1C,D show ramp-induced oscillations in the presence of SB + TTX + MEC before CAR application showing beta frequency oscillations at 25 Hz, and the addition of CAR for 20 min led to a significant increase in oscillation frequency (but not amplitude) to 50 Hz. Superfusion of MOD for 20 min in the presence of CAR significantly increased oscillation amplitude, but decreased oscillation frequency to 30 Hz. These results showed that MOD potentiated CAR-induced oscillation amplitude, but decreased their frequency from the gamma to the beta range in most cells, as was the case with MOD alone (64).

These data suggest that the stimulant MOD preferentially promotes high-frequency activity through the CaMKII (waking) pathway, especially in the presence of tonic cholinergic input. Moreover, studies on cocaine abusers (70), and on an animal model of sleep-disordered breathing (71), suggest that MOD may also decrease REM sleep. That is, MOD may exercise a “push” toward waking while exerting a “pull” away from REM sleep. Further work will be needed to determine if the cAMP/PK (REM sleep) pathway is preferentially activated during REM sleep compared to the CaMKII pathway. These data may also have implications for the mechanisms behind the developmental decrease in REM sleep (68). One possibility is that there is a gradual shift from the cAMP/PKA pathway to the CaMKII pathway with age. This would account for the more abundant generation of gamma band activity in the “waking gamma” compared to the “REM sleep gamma” pathway in the adult compared to the newborn.

## Schizophrenia and Bipolar Disorder

Schizophrenia and bipolar disorder are characterized by sleep–wake symptoms such as hyperarousal, increased REM sleep drive, decreased SWS, and hallucinations (72). Sleep disruptions observed in bipolar patients are state-dependent, showing that manic and depressed patients exhibit shortened total sleep time and REM sleep latency. Although the number of studies from patients in the euthymic period is limited, insomnia and diminished sleep efficiency have been described (73). In fact, hallucinations in these patients have been proposed to represent REM sleep intrusion into waking (74). That is, the states of waking and REM sleep, which are marked by gamma band activity, are disturbed in schizophrenia and bipolar disorder, and contribute to many of the hypervigilance and sleep–wake symptoms observed. These disorders are characterized by decreased or interrupted gamma band activity (75, 76). In postmortem studies, increased neuronal calcium sensor protein-1 (NCS-1) expression was present in schizophrenia and bipolar disorder patients, but not in major depression patients (77, 78). That is, gamma band activity is reduced or disrupted in the two disorders that show brain NCS-1 over expression. We tested the hypothesis that NCS-1 modulates calcium channels in PPN neurons that generate gamma band oscillations, and that excessive levels of NCS-1, as would be expected with over expression, reduce or block gamma band oscillations in these cells.

**Figure 2:**
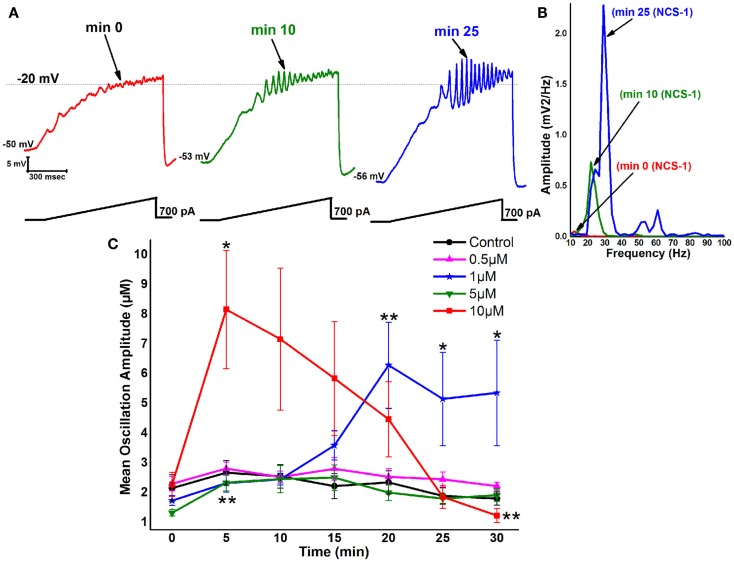
**Effects of NCS-1 on PPN membrane oscillations**. **(A)** Representative 1 s long current ramp-induced oscillations in a PPN neuron in SB + TTX extracellular solution and 1 μM NCS-1 in the recording pipette (left record, red). After 10 min of NCS-1 diffusing into the cell, the oscillatory activity increased slightly (middle record, green). However, after 25 min of NCS-1 diffusion both oscillation amplitude and frequency were increased (right record, blue). **(B)** Power spectrum of the records shown in **(A)** showing the increased amplitude and frequency of oscillations after 25 min exposure to 1 μM NCS-1. (**C)** The graph shows the mean peak amplitude in millivolt of oscillations in control cells recorded (black circles) that demonstrated no significant changes over time. Cells recorded using 0.5 μM NCS-1 also showed no significant changes over time (pink triangles). Cells recorded using 1 μM NCS-1 (blue stars) showed significant increases in mean peak oscillation amplitude at 20–30 min. Cells recoded using 5 μM NCS-1 (green downward triangles) showed no significant changes over time, but cells recorded using 10 μM NCS-1 (red squares) showed a significant increase in mean peak oscillation amplitude at 10 min, but not thereafter. *p < 0.05; **p < 0.01. These results suggest that NCS-1 at low concentrations potentiates beta/gamma oscillations in PPN neurons, but at high concentrations compatible with over expression it reduces or blocks high-frequency membrane oscillations (64, 79).

During recordings in PPN neurons (in the presence of SB + TTX), 1 μM NCS-1 increased the amplitude and frequency of ramp-induced oscillations within ~25 min of diffusion into the cell. Figure 2A is a representative example of ramp-induced membrane potential oscillations in a PPN neuron in the presence of SB + TTX. Shortly after patching, the ramp typically induced low amplitude oscillations in the beta/gamma range. Figure 2A green record shows that, after 10 min of recording, some increase in the oscillation frequency was present (also evident in Figure 2B as a green line in the power spectrum). After 25 min of recording, NCS-1 at 1 μM significantly increased the frequency of oscillations (blue record in Figure 2A and as the blue line in the power spectrum). The graph in Figure 2C shows that control cells manifested no significant changes in amplitude throughout the 30 min recording period. These values were not significantly different from each of the 0 min recordings using pipettes with NCS-1, so that the 0 min recordings are an accurate representation of control levels. When the pipette contained 0.5 μM NCS-1, no changes in amplitude were observed throughout the recording, suggesting that this concentration does not significantly affect oscillation amplitude. When using 1 μM NCS-1, however, the oscillation amplitude increased significantly by 20 min and thereafter, suggesting a gradual effect in tripling amplitude as the NCS-1 diffused into the cell. When using 5 μM NCS-1, there was a significant increase in amplitude at 5 min but not afterward. This effect was probably due to the low amplitude of the initial oscillations in this group of cells. There were no further changes observed, so that we conclude that the effect at 5 min was not consistent. When using 10 μM NCS-1, the oscillation amplitude immediately increased to four times the levels and gradually decreased until it was significantly reduced by 30 min. These effects suggest an immediate effect on amplitude by very high levels of NCS-1 that ultimately led to partial blockade. Based on these results, 1 μM NCS-1 seems to be the most critical concentration for promoting gamma oscillation modulation, although some effects were evident with 0.5 μM NCS-1 (64).

Decreases in gamma band coherence and maintenance can account for many of the symptoms of schizophrenia and bipolar disorder. The positive symptoms include hallucinations, delusions, thought disorder, and agitation, while negative symptoms include lack of affect, anhedonia, and withdrawal. Cognitive symptoms include poor executive function, lack of attention, and disturbed working memory. All these cognitive functions are associated with gamma band activity. The postmortem results previously described (78) suggest that only some patients with schizophrenia may suffer from significant over expression of NCS-1, which may be manifested as decreased gamma band activity only in a subpopulation of patients. No human study has measured gamma band activity and correlated it with NCS-1 levels. Unfortunately, serum sampling does not reflect brain levels and, in fact, NCS-1 levels in leukocytes are actually decreased in schizophrenic patients (80). However, future clinical trials in patients with schizophrenia or bipolar disorder may benefit from prior determination of a significant decrease in gamma band activity, which may also help address the heterogeneity of schizophrenia and facilitate the process of identifying more homogeneous groups within the syndrome (81). It is to those patients that pharmacological targeting to increase gamma band activity may be of benefit. We have preliminary evidence suggesting that the stimulant MOD may indeed compensate to some extent for excessive amounts of NCS-1. We found a partial return of gamma oscillations that were suppressed by high levels of NCS-1 after exposure to MOD (64).

## Leptin, Obesity, and Sleep

Interestingly, obesity is characterized by similar sleep/wake disturbances to schizophrenia and bipolar disorder, such as excessive daytime sleepiness, increased REM sleep, increased nighttime arousals, and decreased percentage of total sleep time (82, 83). Leptin, a hormone that regulates appetite and energy expenditure, is increased in obese individuals, although these individuals often exhibit leptin resistance (84). Several studies have shown that short sleep duration is highly correlated with decreased leptin levels in both animal and human models (85–87). The goal of this project was to determine the role of leptin in the PPN, and thus in obesity-related sleep disorders. Leptin decreased action potential (AP) amplitude, AP frequency, and h-current (I_H_). These findings suggest that leptin causes a blockade of Na^+^ channels. Sodium current amplitude was decreased in a dose-dependent manner, suggesting a direct effect of leptin on these channels. The average decrease in Na^+^ conductance by leptin was ~40% (88). Additional results suggested that the effects of leptin on the intrinsic properties of PPN neurons are leptin receptor- and G protein-dependent. We also found that leptin enhanced NMDA receptor-mediated responses in single neurons and in the PPN population as a whole, an effect blocked by a leptin antagonist (89).

**Figure 3:**
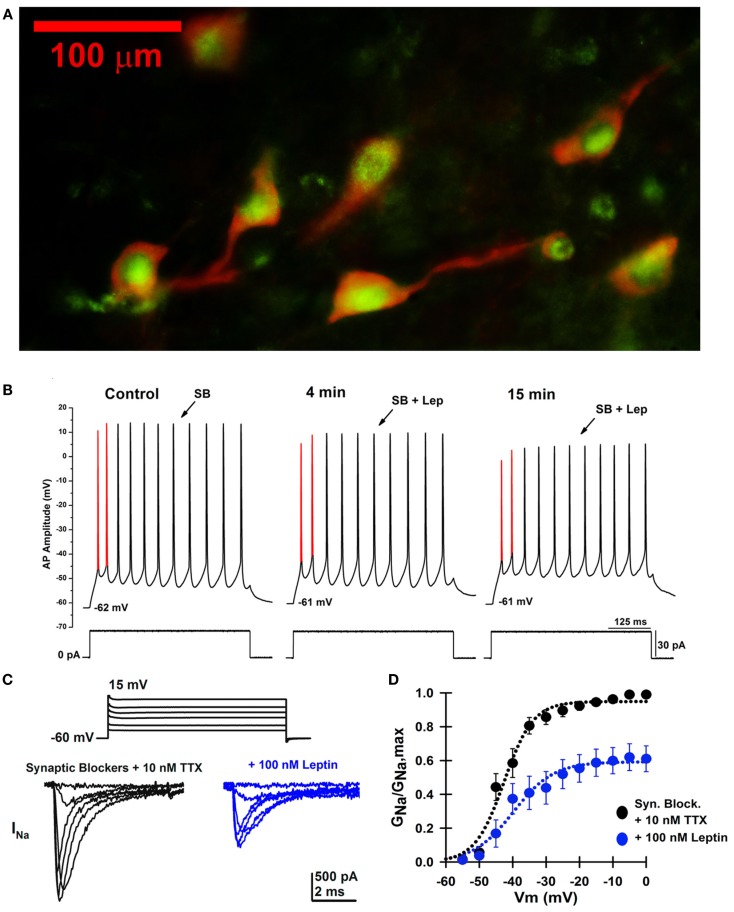
**Effects of leptin on PPN neurons**. **(A)** Photomicrograph for both bNOS and leptin receptor immunohistochemical labeling of a section through the PPN of the rat. bNOS labeled cholinergic PPN cells as solid red cytoplasm (rhodamine filter). Leptin receptor labeling was evident as punctiform green label (FITC filter). This suggests that all or most cholinergic cells in the PPN bear leptin receptors, and that many non-cholinergic cells also bear leptin receptors. **(B)** Whole-cell patch clamp recording in the presence of SB in the same PPN neuron before (left), after 4 min of leptin (100 nM) exposure (middle), and after 15 min of leptin exposure (right). This PPN neuron was subjected to a 30 pA depolarizing current step at all three time points. The first and second APs are highlighted in red from AP threshold to AP peak amplitude. Note the gradual decrease in AP amplitude during the time course, suggesting that leptin reduced sodium currents. **(C)** Whole-cell patch clamp recording in the same PPN neuron in the presence of SB, 0.2 mM CdCl_2_, 0.2 mM NiCl_2_, 20 mM TEA-Cl, and 3–10 nM TTX before (black records) and after 15 min of leptin exposure (100 nM, blue records). The neuron was subjected to voltage steps from −60 to +15 mV. **(D)** Average sodium conductance as a fraction of maximum conductance before (black curve) and after 15 min of leptin exposure (blue curve). These results suggest that leptin partially reduced sodium currents in PPN neurons, which decreases the activation of waking and REM sleep by the PPN (88, 89).

Figure 3A shows a fluorescence photomicrograph of a section through the PPN after processing for bNOS and leptin receptor immunohistochemical labeling. This shows that all PPN cholinergic cells manifested both bNOS and leptin receptor labeling, while non-cholinergic cells showed only leptin receptor labeling evident as green punctiform label without bNOS labeling. This suggests that all cholinergic cells in the PPN bear leptin receptors, and that many non-cholinergic cells also bear leptin receptors. Figure 3B is an example of a single cell current clamp recording before and during administration of leptin showing a decrease in AP amplitude after leptin. Figures 3C,D show that leptin reduced sodium currents in PPN cells.

We hypothesize that leptin normally acts through G proteins to decrease activity in the PPN by reducing *I*_H_ and *I*_Na_ currents, and that in states of leptin dysregulation (i.e., leptin resistance) this effect may be blunted. Such an effect would lead to increased arousal and REM sleep drive, and ultimately to sleep-related disorders such as those seen in obesity (88, 89).

## Cell Clusters

Recent studies determined that in the hippocampus there are, according to one group, “patches” of entorhinal cortex cells that play a role in learning and memory (90). Another group described clusters of cells that may have joint function as “islands” that form a hexagonal lattice over the cortex (91). Are there clusters of cells that act together in the PPN to modulate coherence and frequency? The answer is that we do not know, however, there is intriguing evidence to suggest that the PPN may contain subgroups of functional units.

Anatomically, neurons in the PPN are scattered such that in the *pars compacta* there are intermingled glutamatergic, cholinergic, and GABAergic neurons in the ratio of 5:3:2, respectively (92). Studies using calcium imaging in the PPN *pars compacta* reveal an interesting anatomical organization within the nucleus. Since electrically coupled neurons generally represent GABAergic neurons, we speculate that there are five glutamatergic and three cholinergic neurons closely associated with each GABAergic pair. That is, there may be clusters of 10 neurons scattered within the *pars compacta* that may create a functional subgroup. Much additional evidence is required to support this hypothesis, but it may be possible to dissect such an organization to determine how the nucleus as a whole generates coherent activity at specific frequencies. It is also important to determine how PPN neurons respond to sensory input and how that input generates coherent activity. Similar functional clustering has been proposed for the hippocampus, especially in relation to epileptic networks (93). In a study of cell clusters in the hippocampus, Buszaki described subsets of about 10 neurons that showed repeated synchronous firing during open field exploration (94). Interestingly, the time scale of activity between these neurons had a median of 23 ms, and the peak optimal timescale was ~16 ms, that is, most activity occurred in the 30–60 Hz range. We hypothesize that a similar temporal relationship is evident among cell clusters in the PPN.

Do brain circuits “at rest” have a natural frequency at which they resonate? Using sensory stimulation such as light flashes or auditory tone stimuli, various workers have found a thalamocortical resonant frequency ~10 Hz in human beings and animals (95–97). While these responses assess the tuning of thalamocortical circuits, they do so indirectly because they test the pathway that includes peripheral receptors, intervening synapses, induced sensory gating, changes in attention levels. Others have used direct perturbations to detect the main rate of ensuing oscillations, or the natural frequency of cortical regions (98). These authors used transcranial magnetic stimuli to perturb different cortical regions directly in order to measure their natural resonant frequency. We undertook parallel studies on the PPN. We used sagittal slices containing the rodent PPN and recorded voltage-sensitive dye population responses using ANEPPS.

**Figure 4:**
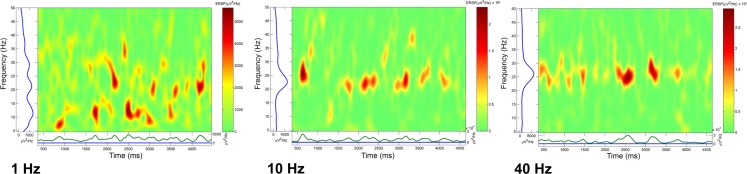
**Natural frequency of PPN population**. Event related spectral perturbations (ERSPs) calculated after loading the slice with the voltage-sensitive dye ANEPPS. In order to increase background activity, carbachol (CAR) was perfused (30 μM) and the slice stimulated using a bipolar electrode inserted at the posterior edge of the PPN. The ERSPs represent a rolling power spectrum over time after delivery of the stimulus of four pulses. The train of four stimuli was delivered at 1 (left ERSP), 10 (middle ERSP), and 40 Hz (right ERSP). Note the increasing coherence as stimulation frequency increased until high amplitude bursts of activity were induced for 4–5 s following the stimulus. These studies suggest that the natural frequency for inducing maximal coherence was in the gamma band range (1).

We calculated event related spectral perturbation (ERSP) that represents average dynamic changes in amplitude of the broad band EEG frequency spectrum as a function of time relative to an experimental event (99). We calculated ERSPs after delivering trains of four stimuli at 1, 10, or 40 Hz, and recorded population responses for several seconds following the last stimulus of each train. In the absence of activation with specific transmitters, these stimuli did not produce significant effects, therefore, we superfused CAR in order to raise the overall excitability of the slice. Figure 4 shows that stimulation using a four-pulse train at 1 Hz (left ERSP) induced bursts of activity at various frequencies. When the four-pulse train was delivered at 10 Hz, there was more consolidation of the response with less variability that at 1 Hz. When the four-pulse train was delivered at 40 Hz, what resulted was highly synchronized activity recurring at beta/gamma frequencies (1). These studies suggest that the natural frequency of the PPN population may be at beta/gamma frequencies, in keeping with the characteristics described above in terms of calcium channel-dependent subthreshold gamma oscillations.

## Conclusion

The PPN generates and maintains beta/gamma band activity upon waking and during REM sleep. It does so through membrane oscillations mediated by voltage-dependent high threshold N- and P/Q-type calcium channels that are modulated by G proteins. In addition to intrinsic membrane oscillations, the maintenance of gamma band activity requires synaptic connectivity within the nucleus and between regions of the brain. PPN intranuclear circuitry includes cholinergic, glutamatergic, and GABAergic neurons. Some GABAergic cells are electrically coupled to provide coherence, and the nucleus may include functional cell clusters. From the moment we awaken, this mechanism ensures that the necessary background of activity is present in order to preconsciously evaluate the world around us. Therefore, this process is embedded in the formulation of our perceptions and actions, and modulates higher-level beta/gamma processing through its projections to the intralaminar thalamus, basal ganglia, hypothalamus, and basal forebrain. That is why it affects functions as disparate as waking and REM sleep, mood and perception, and homeostatic regulation. Consequently, dysregulation in PPN processing will be manifested in psychiatric disorders, neurological disease, as well as sleep disturbances.

## Future Directions

The PPN has become a target for DBS for the treatment of movement and postural disorders in patients with PD. A recent review describes the effects on posture, gait, sleep, and cognitive function observed following PPN DBS (21). Such use was predicted almost 30 years ago based on animal studies using stimulation in the region of the PPN, which at the time was thought to be in the mesencephalic locomotor region (MLR) (100–102). The MLR was originally described as a region that, when stimulated with increasing current amplitudes using long duration (0.5–1.0 ms) pulses at 40–60 Hz in the precollicular–postmamillary transected, weight-suspended cat, would induce controlled locomotion on a treadmill (103). Novel results described in the PPN help explain why, in order to induce locomotion, (a) lateral, but not medial, cuneiform nucleus stimulation was required, (b) ramping up of the current was required, (c) stimulation at 40–60 Hz, but not higher or lower, was required, and (d) long duration pulses were required (64). A potentially fruitful future endeavor is to determine the effects of PPN DBS on gamma band power. A number of neurological and psychiatric disorders are characterized by interrupted or decreased gamma band activity (21). Determining the effects of DBS in potentiating gamma band power and on gamma band maintenance would be very important for assessing the beneficial effects of PPN stimulation on higher functions.

Another potentially fruitful endeavor is the study of the medial cholinergic cell group known as the laterodorsal tegmental nucleus (LDT). While the LDT is more compact and easier to localize, the fact remains that stimulation of the LDT does not induce changes in muscle tone or locomotion like stimulation of the PPN (101, 104), i.e., may not participate in the motor components of adult waking and REM sleep. Moreover, its ascending projections do not help generate arousal-related evoked potentials (105, 106), and ascending LDT projections are directed at more “limbic” regions (ventral tegmental area, basal forebrain, accumbens) than those of the PPN, which are more “motor” (substantia nigra, intralaminar thalamus, striatum) (101, 104–106). Certainly, based on its connectivity, the LDT could not be considered a target for DBS for the alleviation of motor deficits in PD, and has typically not been investigated for its ability to modulate intralaminar thalamus and cortical high-frequency EEG. However, its location within the central gray and its projections to the ventral tegmental area that is a way station to the accumbens and limbic system, would suggest that the LDT may respond to painful stimuli, or at least afferents with “affective” properties. Assuming, just because the neuronal complements and cellular properties are similar to those of PPN neurons that the LDT has a similar function in waking and arousal is unsubstantiated. Better directed investigation of the LDT than that published to date is needed.

## Conflict of Interest Statement

The authors declare that the research was conducted in the absence of any commercial or financial relationships that could be construed as a potential conflict of interest.
